# Effects of Graphene Oxide and Crumb Rubber on the Fresh Properties of Self-Compacting Engineered Cementitious Composite Using Response Surface Methodology

**DOI:** 10.3390/ma15072519

**Published:** 2022-03-29

**Authors:** Isyaka Abdulkadir, Bashar S. Mohammed, Montasir Osman Ahmed Ali, M. S. Liew

**Affiliations:** 1 Civil and Environmental Engineering Department, Faculty of Engineering, Universiti Teknologi PETRONAS (UTP), Bandar Seri Iskandar 32610, Perak, Malaysia; isyaka_18000638@utp.edu.my (I.A.); montasir.ahmedali@utp.edu.my (M.O.A.A.); shahir_liew@utp.edu.my (M.S.L.); 2 Civil Engineering Department, Bayero University, Kano 700241, Nigeria

**Keywords:** graphene oxide (GO), engineered cementitious composite (ECC), crumb rubber (CR), self-compacting ECC

## Abstract

Graphene oxide-modified rubberized engineered cementitious composite (GO-RECC) is attracting the attention of researchers because of the reported benefits of the GO and crumb rubber (CR) on the strength and deformation properties of the composite. While it is well established that GO negatively affects the workability of cementitious composites, its influence on the attainment of the desired self-compacting (SC) properties of ECC has not yet been thoroughly investigated, especially when combined with crumb rubber (CR). In addition, to simplify the number of trial mixes involved in designing SC-GO-RECC, there is a need to develop and optimize the process using Design of Experiment (DOE) methods. Hence, this research aims to investigate and model using response surface methodology (RSM), the combined effects of the GO and CR on the SC properties of ECC through the determination of T_500_, slump flow, V-funnel, and L-box ratio of the SC-GORECC as the responses, following the European Federation of National Associations Representing for Concrete (EFNARC) 2005 specifications. The input factors considered were the GO by wt.% of cement (0.02, 0.04, 0.06, and 0.08) and CR as a replacement of fine aggregate by volume (5, 10, and 15%). The results showed that increasing the percentages of GO and CR affected the fresh properties of the SC-GORECC adversely. However, all mixes have T_500_ of 2.4 to 5.2 s, slump flow of 645 to 800 mm, V-funnel time of 7.1 to 12.3 s, and L-box ratio (H2/H1) of 0.8 to 0.98, which are all within acceptable limits specified by EFNARC 2005. The developed response prediction models were well fitted with R^2^ values ranging from 91 to 99%. Through the optimization process, optimal values of GO and CR were found to be 0.067% and 6.8%, respectively, at a desirability value of 1.0.

## 1. Introduction

Current infrastructure development trends push conventional construction materials to their limits and urge researchers to develop new materials or improve existing ones to meet the constructions’ needs [1,2,3]. One such quest for improved materials yielded engineered cementitious composite (ECC). ECC, also known as bendable concrete, is a highly ductile composite with a strain capacity of 3–8% with the ability to withstand loads above the initial cracking stress and exhibit strain hardening behavior [4,5]. This behavior is attributed to the composite’s ability to develop and propagate saturated steady-state microcracks with a width of less than 100 μm. It is achieved by utilizing the micromechanics principles in the design of the composite to modify the interaction of the cement matrix, polymeric fibers, and their interface [6,7,8].

ECC is mainly utilized for repair and retrofitting works, although many attempts are being made to improve its properties for structural purposes [4,9,10]. In this regard, researchers have discovered that incorporating CR in ECC improves energy absorption, enhances microcrack formation and ductility, and leads to better durability properties [4,5,11,12,13,14,15]. In addition, CR is reported to prevent explosive spalling of ECC at elevated temperatures by melting and providing escape routes for the vapor pressure. Furthermore, the use of CR in ECC and other cementitious composites partially contributes to eradicating the menace of waste tire disposal, which constitutes a considerable part of the global waste management challenges faced in the 21st century [5,16,17]. However, CR is reported to weaken the mechanical properties of cementitious composites [18,19,20]. This is due to the rubber particles’ reduced stiffness and the weak bonding with the hardened cement paste [9,21,22]. To overcome the disadvantage of CR’s inclusion, nanomaterials such as nano-silica and graphene oxide (GO) have been added to the rubberized ECC mixtures to enhance the bonding between CR particles and hardened cement matrix and consequently, to improve the strengths of the hardened RECC [1,21,23,24].

Although research works on using GO in ECC and rubberized ECC (RECC) are still in the early stages, the preliminary findings are encouraging due to its superior performance over other nanomaterials. GO has a 2D planar structure with numerous oxygen-containing functional groups on its surface [25]. The platelet structure enables it to physically intercept crack growth at the nano level in addition to the pore filling effect, unlike 0D nanomaterials such as nanosilica, which has only a filling effect at the physical level. Because of the abundance of the hydrophilic functional groups on the GO’s basal planes, it is easier to disperse in the mix, enhancing its effectiveness, unlike 1D nanomaterials such as carbon nanotubes, whose high interparticle cohesive force hinder proper dispersion [26]. These advantages make GO a popular choice in the nanomodification of cementitious composites, with encouraging reported findings.

GO is increasingly being used in the modification of RECC. Sebapathy et al. [12] reported a significant increase in the compressive strength of RECC with an increase in GO content. There was a 19.2% increase in the compressive strength between a mix having 0.01% GO and that with 0.05% GO at a 10% CR replacement level. Similarly, Hau Hong et al. [8] reported a 14% and 48% increase in the compressive strength and modulus of elasticity between a mix having 0.01% GO content and one with 0.05% GO, both at 10% CR replacement levels. This is due to the GO’s high reactivity, which speeds up the hydration reaction, resulting in increased calcium-silica-hydrate (C-S-H) gel formation and the pore-filling effect, which densifies the microstructure of the composite. The densification of the microstructure through the pore-filling effect of additives also improves the durability of the composite [27]. Furthermore, pretreating CR with GO has been reported to reduce the strength loss and enhance the mechanical properties of the composite, which was caused by the GO sticking to the CR surface, lowering the rubber particles’ hydrophobicity, and improving the bonding with the hardened cement paste [10]. In addition, GO is reported to reduce the drying shrinkage of rubberized ECC [1] due to the pore filling effect and the refinement of the microstructure, which reduced the composite’s volumetric change tendency caused by drying shrinkage. Abdulkadir et al. [9] also reported an improvement in rubberized ECC’s impact resistance and energy absorption due to the enhanced bonding between the CR and the cement matrix.

ECC is generally designed to have self-compacting (SC) properties in the fresh state. ECC designed as self-compacting (SC-ECC) in a fresh state has adequate flowability, passing ability, filling ability, and resistance to segregation [24,28]. Previous research has reported that adequate mix flowability and stability are necessary for proper fiber dispersion, which is critical in achieving the fibers’ crack-bridging effect, translating into the ECC’s exceptional ductility and strain-hardening behavior [29]. Similarly, an SC-ECC provides higher packing density and improved performance parameters due to its pumpability and packing density [30]. Obtaining a particularly flowable ECC mix comprising GO and CR is difficult due to their flowability-reducing effect.

The negative effect of GO on the fluidity of cementitious composites has been fully established. A recent finding by Wei et al. [31] revealed a decrease of 7.9%, 10.0%, and 13.1% in the fluidity of fly ash—cement mortar containing 0.01, 0.02, and 0.03 wt.% of GO, respectively. In the same vein, Lee et al. [32] reported a decrease in the slump of cementitious composite by 22.7–45.5% with the addition of GO. The main reason given is that GO’s hydrophilic functional groups and large surface area allow it to adsorb a considerable amount of water during mixing. As a result, the amount of water required to lubricate the cement grains is lowered, increasing the interparticle friction. Hence, GO increases the viscosity of the mix, which in turn reduces its fluidity and workability [32,33]. Similarly, researchers have reported that the inclusion of CR in ECC mixes negatively affects the workability properties of the mix [16,34,35,36]. Ismail et al. [37] reported a 43.4 and 54.6% increase in rubberized ECC’s T_500_ and V-funnel times, indicating lesser mix workability with CR increasing from 0 to 30%. They attributed this to the increasing volume of larger-sized CR particles, which increased interparticle friction due to their rough surface texture, affecting the capacity of the mix to flow under its weight. However, unlike GO, where its reducing effect on the fluidity is unanimous, some researchers have reported an increase in ECC’s workability with an increase in CR, attributed to the hydrophobic nature and water repulsion effect of the rubber particles, leading to an increase in the amount of water in the mix [38,39]. Hence, this calls for more research on the effect of CR on the workability of SC-ECC.

With the present surge of interest in GO-RECC research, there is scarce information on the combined effects of GO and CR on SC properties, especially given the reported adverse effect of these materials on the workability of the mix separately. Most researchers focus more on the hardened properties, neglecting the material’s performance in the fresh state, which is essential in attaining the desired properties in the hardened state. Therefore, there is a need to get optimized levels of these additives to guarantee the desired SC performance in the fresh state.

SC mix optimization generally involves multiple trial batches to balance flowability, stability, and hardened properties [29,40]. This process becomes more tedious with an increased number of variables, such as in the case of SC-GO-RECC. To make this process easier, a suitable experimental design procedure is required to develop an SC-GO-RECC that performs as expected in fresh and hardened stages. The following are some of the benefits of using an experimental design method: (i) the development of an empirical response predictive model using the input variables; (ii) reducing the number of experiment and trial batches; (iii) the assessment of the interaction between the different variables; and (iv) the determination of the optimal levels of the input variables and the response within the design space [29]. This justifies the purpose of this work.

One of the most commonly used experimental designs is the response surface methodology (RSM). RSM is a set of statistical and mathematical tools for designing, enhancing, and optimizing processes [41]. It is also helpful in designing, developing, and formulating new products and enhancing existing product designs [42,43,44]. It involves determining the impact of some selected input factors (in this instance, the GO and CR) and their interaction on the response(s) of interest, also known as the dependent variable(s) or output factor(s) (the SC properties in this case). Some researchers have used RSM to model the SC performance of some types of cementitious composites (CCs), such as self-compacting high volume fly ash ECC [43], SC-hybrid fiber-reinforced rubberized CC [7], ultra-high-performance concrete reinforced with micro-steel fibers [29]. However, no such approach has been used in the case of SC-GO-RECC previously; thus, the goal of this study is to use the RSM to determine, model, and to optimize the impacts of GO and CR on the SC characteristics of ECC based on the National Associations Representing for Concrete (EFNARC) 2005 requirements.

## 2. Materials and Methods

### 2.1. Materials

**Figure 1:**
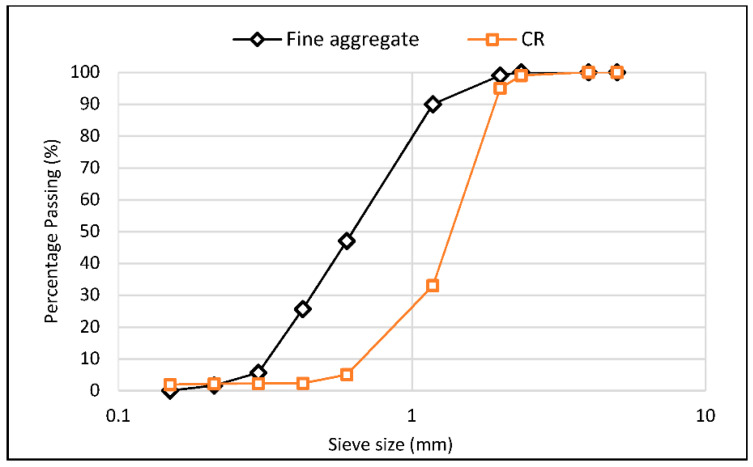
Grading curve for fine aggregate and CR used.

The materials used to prepare the mixtures include type I ordinary Portland cement (OPC) of grade 32.5R, satisfying the Malaysian standard MS EN 197-1 [44]. Fly ash (FA) having a total (SiO_2_ + Al_2_O_3_ + Fe_2_O_3_) oxide content of more than 70% and classified as F based on ASTM C618 specifications was used. The FA has a specific gravity, fineness, and loss on ignition of 2.3, 1.5895 m^2^/g, and 1.87%, respectively. River sand with an average particle size of 400 μm, a specific gravity of 2.65, and a fineness modulus of 2.20 was used as the fine aggregate. The CR used for partially replacing fine aggregate has particles passing 1.18 mm sieve and a specific gravity of 0.95. Figure 1 shows the grading for the fine aggregate and the CR. The fiber used was a polyvinyl alcohol fiber (PVA) with 1.2 wt.% oil coating. The coating guards against excessive bonding between the fiber and the cement paste, resulting in fiber rupture under loading. The fiber has a length of 18 mm, a diameter of 40 μm, a specific gravity of 1.3, a tensile strength of 1600 MPa, and an elastic modulus of 41 GPa. Graphene oxide (GO) used is in a very viscous state of 2.5 wt.% concentration. In order to ensure adequate workability of the mixtures, a superplasticizer (SP) was used, having a PH of 6.2, specific gravity of 1.08, and 0.2% chloride ion content.

### 2.2. RSM Variables and Mix Proportion

The RSM package on Design-Expert Software (Version 10) was used for the experimental design, modeling, and optimization. The procedure is divided into three steps, which are as follows: (1) generating and running a series of experiments using different combinations of input factor values. The experimental runs can be generated using different design options, including central composite design (CDD), Box Behnken Design (BBD), User Defined Design (UDD), and Optimal Design (OD). (2) Based on the level of interaction of the independent and dependent variables, mathematical response predictive models are developed based on the empirical data gathered in the first step. The developed models are then validated using analysis of variance (ANOVA). Lastly, (3) a multi-objective optimization is used to find the best solution for the levels of the input factors and the responses under consideration. In order to assess the outcome of the optimization and the prediction of the model’s strength, experimental validation is usually conducted [6,22,45].

In this case, the input variables were the GO at 5 levels (0, 0.02, 0.04, 0.06, and 0.08 wt.%) and CR partial replacement of fine aggregate at 3 levels (5, 10, and 15% by volume). Fifteen experimental runs (mixes) were generated using the RSM, as shown in Table 1. Compared to CCD, BBD, and OD options, the UDD option of the RSM was chosen since it offers greater freedom and flexibility in terms of the number of levels of the variables at significantly lower experimental runs. The responses considered were the workability properties assessed through the slump flow, T_500_, V-funnel, and L-box tests based on the requirements of EFNARC 2005 [46]. For comparison purposes, a control mix (NECC) of normal ECC-M45 [47,48,49] without GO (0% GO) and CR (0% CR) was produced and tested for all the workability properties.

### 2.3. Mixing and Testing Procedure

#### 2.3.1. Mixing

**Figure 2:**
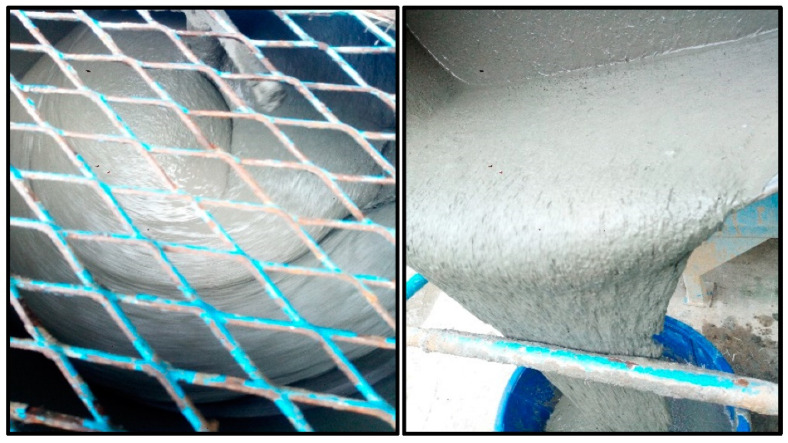
Freshly mixed SC-GORECC.

The dry materials, including cement, FA, fine aggregate, and CR, were initially mixed for two minutes in a pan-type concrete mixer with double rotation capability. Following that, a mixture of water, HRWR, and GO was added to the mixer and mixed thoroughly for 5 min. The GO was well dispersed in the mixing water due to the dispersing effect of the polycarboxylate-based superplasticizer as established in previous studies [34,50,51]. PVA was gradually added to the mix for 5 min through the mesh window on top of the mixer to guarantee fiber dispersion and avoid fiber balling. The mixing was maintained for another 5 min to obtain a visually uniform and homogeneous blend, as shown in Figure 2.

#### 2.3.2. Workability Tests

As described by EFNARC, freshly mixed concrete is self-compacting only if it satisfies the filling ability, passing ability, and segregation resistance requirements [35]. These requirements are determined through the slump flow (with Abram’s cone), V-funnel, and L-box tests.

**Figure 3:**
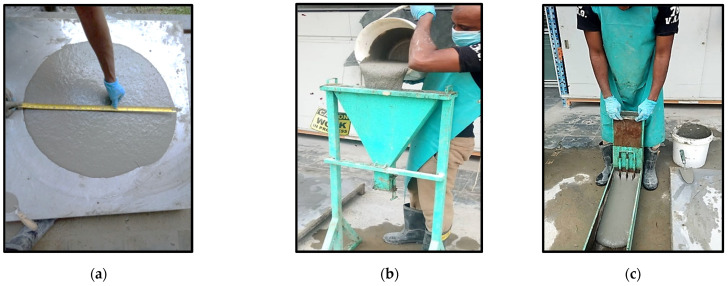
(**a**) Slump flow test; (**b**) V-funnel test; (**c**) L-box test.

For slump flow and T_500_, the Abrams (slump) cone has been used to assess the ability of fresh SCC to flow. The fresh ECC has been poured into the slump cone placed on a nonabsorbent platform marked with two concentric circles of 500 mm and 1000 mm diameters. The slump flow was measured using the average flow diameter measured from two orthogonal directions, as depicted in Figure 3a.

T_500_ is a secondary measure of flow. Once the slump cone is removed after filling with the fresh concrete, the time taken for the mixture to reach a 500 mm spread circle is recorded as T_500_ flow time. The V-funnel test was used to determine the self-compacting ECC’s filling-ability. The test was carried out by filling the v-shaped funnel with fresh ECC, as shown in Figure 3b, and timing how long it took to flow out through the bottom opening.

The L-box test assesses the passing ability of the mixture to pass through the obstacle without the separation of the constituents, as shown in Figure 3c. The test is done using the L-shaped apparatus having two horizontal and vertical segments separated by a sliding gate with three vertical bars. The height of material in the horizontal section of the box (H2) to the height in the vertical section of the box (H1) gives the blocking ratio, which measures the ease of fresh concrete flow.

## 3. Results

### 3.1. T_500_

**Figure 4:**
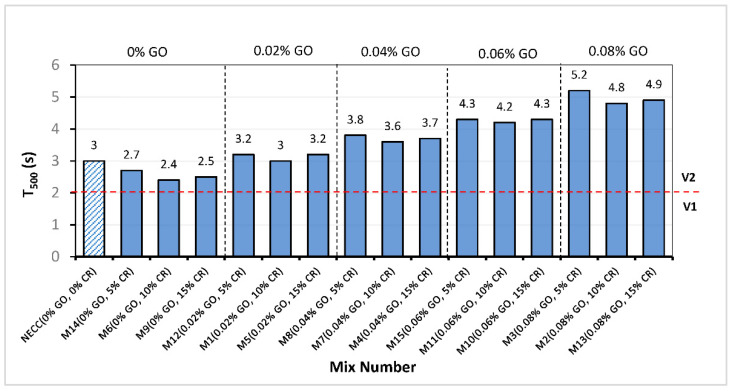
T_500_ for all mixes.

The T_500_ result of all the mixes is shown in Figure 4. This test measures the flow rate of the fresh SC-GO-RECC, and it helps assess the viscosity of the mix. The measured time does not directly give a measure of viscosity but is related to it as concrete with low viscosity will flow fast for a short time and then stop, while that with low viscosity will flow gradually over an extended time [46]. As shown in Figure 4, EFNARC classified the degree of viscosity into two categories (V1 and V2). All the mixes have a T_500_ of more than 2 s, placing them in the V2 viscosity class. Higher time indicates higher viscosity, which is inversely proportional to the workability of the mix. The trend reveals that as GO levels increased, the T_500_ increased by 92.6% between M14 and M3, both of which have 5% CR but contain 0% and 0.08% GO, respectively. A similar increase in the viscosity of the mixes with an increase in the level of GO can be observed across all the mixes having the same CR replacement levels. This is attributed to the high affinity between the surface functional groups on the GO and the cement particles, which increased the flocculation of the cement particles trapping free water, aggravating the loss in the fluidity of the mix [33].

In addition, it can be observed that at 0% GO, an increase in CR caused a reduction in the viscosity compared to the control mixture. The T_500_ has a 10, 20, and 16.67% drop for 5, 10, and 15% CR replacements compared to the control mixture. This is due to the hydrophobic CR particles’ water repulsing effect, leading to more fluidity of the mixtures because of the free available water [39]. It is noteworthy that at all GO addition levels, when the CR increased from 15 to 10%, the T_500_ decreased, signifying a reduction in the viscosity of the mixes. This trend occurs because, at a CR replacement level of 5%, it was too low to induce any substantial improvements in workability other than those caused by the GO, and it is entirely dominated by the cement and GO flocculants. However, the GO had a reduced impact at a higher CR level of 10%, which resulted in the excess CR trapping air on their surface owing to their hydrophobic nature [5,52,53], increasing the air content of the mix and, as a result, there was an increased fluidity due to the smooth air bubbles similar to the effect in air-entrained concrete [7].

When the CR replacement was raised to 15%, the viscosity of the mixes increased slightly across all GO levels. This is evident from the slight rise in the T_500_ of all mixes when the CR increased from 10% to 15%. This is due to an increase in interparticle friction caused by the rubber particles’ rough surface morphology [37], which slowed down the spread of the mix to the 500 mm circle.

### 3.2. Slump Flow (SF)

**Figure 5:**
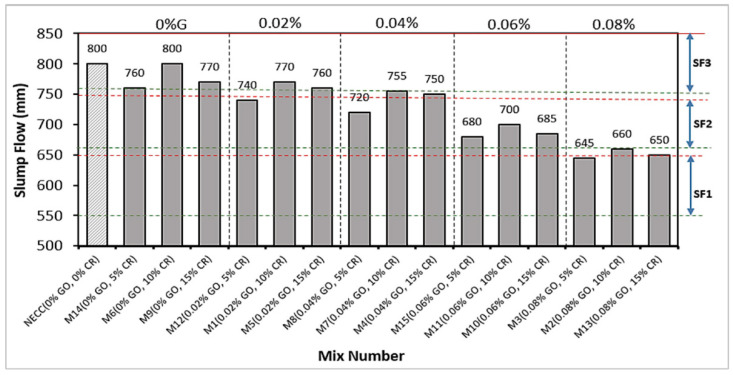
Slump flow values of SC-GO-RECC.

The slump test measures the consistency of a fresh mix. EFNARC classifies the consistency into three: SF1 (500–650 mm), SF2 (660–750 mm), and SF3 (760–850 mm). The result of the SF for the mixes is shown in Figure 5, with green and red lines indicating the lower and upper limits of the SF classifications, respectively. Six of the mixes (M14, M6, M9, M1, M5, and M7) are classified as SF3, six (M12, M8, M4, M15, M11, M10, and M2) as SF2, and just two (M3 and M13) are classified as SF1. The SF of all mixes is between 645 and 800 mm, which is within EFNARC’s acceptable SF limits for SCC. It can be observed that an increase in the GO led to a reduction in the SF. All mixes having no GO (0% G) exhibited a high SF, making them fall within the SF3 class. On the other hand, mixes containing 0.06 and 0.08% GO have the lowest SF values and are classified as SF2 and SF3, respectively. As the GO increases, the gravitational shearing stress required to overcome the yield stress of the mix decreases, thereby reducing the ability of the mix to flow freely [54]. This is due to the GO lowering the amount of free water in the mix to hydrate its large surface area. Furthermore, it has been shown that the negatively charged GO particles get attracted to the cement grains due to electrostatic force leading to flocculation and aggregation, which trapped water and reduced the fluidity of the mix [33,54]. This prevents the mix from spreading as the viscosity is increased.

The effect of CR on the SF follows the same pattern as its effect on T_500_. Compared to the control mixture at 0% GO, 5% CR replacement led to a reduction of 5% in the slump flow. However, as the CR increased to 10%, the loss in the SF was completely recovered. When the CR increased to 15%, the SF reduced by about 4%. The fluctuation in the SF with an increase in the CR from 5% to 15% was observed across all GO additions. The rough-surfaced rubber particles cause greater interparticle friction, which reduces the flowability of the mix at 5% CR. Nonetheless, at 10%, the higher air content of the mix owing to trapped air by the hydrophobic CR boosted flowability due to the trapped air bubbles’ ball-bearing action. When the CR was raised to 15%, however, the low density of the rubber particles hampered the mix’s capacity to flow under its weight, lowering the SF [5].

### 3.3. V-Funnel (VF)

**Figure 6:**
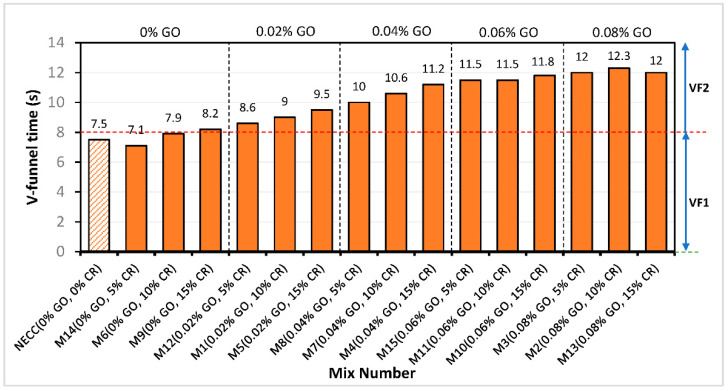
V-funnel time.

Figure 6 shows the V-funnel time of all the mixes. This test also helps in assessing the viscosity of SCC mixes [39]. Based on the EFNARC classification, VF time of zero to 8 s is VF1, while from 9 to 25 s is VF2. Hence, all of the mixes without GO fall into the VF1 class, whereas all the other mixes with GO are in the VF2 class. The VF time increased steadily with an increase in the GO content. At 0% GO, there is a slight reduction of 5.3% at 5% CR replacement. This reduction in the viscosity can be attributed to the hydrophobic effect of the CR, repelling water, making it available for more fluidity and reduced viscosity [7]. However, this effect was lost as the CR replacement increased. When the CR increased to 10 and 15%, the VF time increased by about 5% and 9% compared to the control mixture. The increase in the VF time signified the rise in the viscosity of the mix [24], attributed to the increased interparticle friction due to the rough surface texture of the rubber particles. The result showed that by increasing the CR, the viscosity of all the mixes increased at all GO additions. The highly reactive GO contributes to the mix’s cohesiveness, causing it to have a higher viscosity and flow more slowly than mixes with less or no GO. Similarly, the density of the mix affects the VF flow, which might have been impaired by increasing low-density CR particles, extending the time it takes for the mix to flow out due to gravity.

According to EFNARC guidelines, the VF1 viscosity class mixes may be used for floors and slabs, while the VF2 viscosity class mixes can be used for ramps, walls, and piles.

### 3.4. L-Box Test (H2/H1)

**Figure 7:**
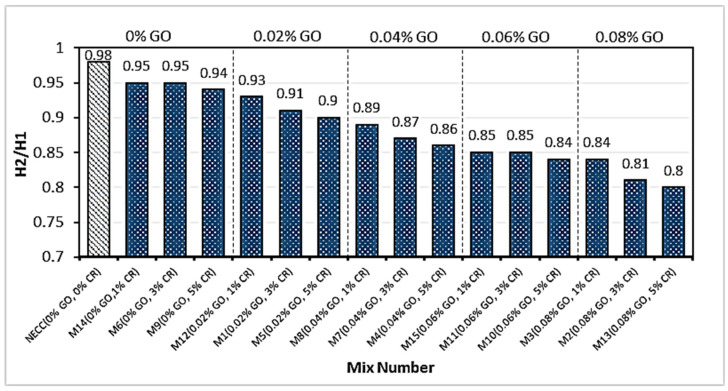
Blocking ratio (H2/H1).

The L-box test measures the passing ability of a mix defined by EFNARC as the ability of the fresh mix to move into confined spaces and narrow channels, such as sections of congested reinforcement, without segregation, loss of homogeneity, or creating a blockage. The passing ability is expressed as a blocking ratio of H2/H1. H1 and H2 are the mix heights in the vertical and horizontal components of the L-box, respectively. The blocking ratio of the mixes follows a downward trend with an increase in the GO addition and CR replacement levels of fine aggregate, as shown in Figure 7. The blocking ratio of all the mixes ranges between 0.8 and 0.98, which is well within the range of values specified by EFNARC.

The reduced passing ability of the mixes with an increase in the variables is due to the increased viscosity. The GO also increased the adherence of the CR and PVA fibers to the cement paste [10,55], making the mixture more viscous and reducing flow and blocking ratios. In contrast to the control mix (NECC), the PVA fibers that ran across the three smooth bars at the flow gate generated more flow resistance, especially at increased GO concentrations.

### 3.5. RSM Models and Analysis of Variance (ANOVA)

The predictive response models in this study were developed using RSM. The models’ reliability was checked using analysis of variance (ANOVA). Response models produced might be linear or higher-degree polynomials as presented in a generalized format in Equations (1) and (2), respectively.

(1) $$y=\beta_{0}+\beta_{i}x_{i}+\beta_{2}x_{2}+\beta_{n}x_{n}+\epsilon$$

(2) $$y=\beta_{0}+\overset{k}{\underset{i=1}{\sum }}\beta_{i}x_{i}+\overset{k}{\underset{i=1}{\sum }}\beta_{ii}x_{i}^{2}+\overset{k}{\underset{j=2}{\sum }}\overset{j=1}{\underset{i=1}{\sum }}\beta_{ij}x_{i}x_{j}+\epsilon \;$$

where y denotes the desired response, $\beta_{0}$ is the regression coefficient for the constant term, whereas $\beta_{i}$, $\beta_{ii}$, and $\beta_{ij}$ are the coefficients for linear, quadratic, and the interaction of $x_{i}$ and $x_{j}$ terms, respectively. The number of factors is denoted by *k*, while the random error is denoted by ϵ.

The response models developed in coded factors are presented in Equations (3)–(6) for the T_500_, SF, VF, and H2/H1. These models are based on empirical data collected from the experiments. High values of the variables are encoded as +1 by default, whereas low levels are encoded as −1. By comparing the coefficients of the variables, the coded equations may be employed to determine their relative importance.

(3) $$T_{500}=+3.72+1.31\times A+0.41\times B+0.01\times \mathrm{AB}+0.031A^{2}+0.23B^{2}$$

(4) $$\mathrm{SF}=+690.67-56.00\times A-21.50\times B-2.00\times \mathrm{AB}-13.33\times A^{2}+1.50B^{2}$$

(5) VF=+11.03+1.81×A+0.62×B−0.53×AB

(6) H2H1=+0.88−0.065×A−0.012×B

A and B denote the GO addition in wt.% of cement and CR replacement of fine aggregate by volume as the independent variables, respectively. It can be seen that the T_500_ and slump flow were fitted with quadratic models while 2FI and linear models were found more suitable for the V-funnel and H2/H1, respectively.

ANOVA test is the next stage in developing RSM-based models, performed for validation. The analysis in this study was done at a 95% confidence level, signifying a probability of 5% (0.05). Thus, any model or model-term with a probability lower than 5% is considered statistically significant. The summary of the analysis is presented in Table 2. As can be observed, all the developed models have a probability of less than 0.05 and are hence significant. The significant terms in the T_500_ and SF models were A, B, and A^2^, showing that the independent variables (A and B) directly influenced the responses and that the responses had a quadratic effect with A, respectively. In the case of the VF model, A, B, and AB are significant model terms signifying that both the input factors directly influence the response, and so was their interaction. A and B are all significant in the H2/H1 model.

The model validation parameters are presented in Table 3. As can be seen, all the developed models have a high coefficient of determination (R^2^) of 99, 98, 90, and 98%, indicating that the models fit the data very well. Furthermore, it is recommended that for a model to fit, the difference between the Adjusted and Predicted R^2^ (Adj. R^2^ and Pred. R^2^) should not be more than 0.2. As can be observed, the difference between the Adj. R^2^ and Pred. R^2^ for all four models is in good agreement, with a difference of less than 0.2. In addition, for a good model, the signal-to-noise ratio indicated by the Adequate Precision value (Adeq. Presc.) should be more than 4, a condition satisfied by all the developed models.

**Figure 8:**
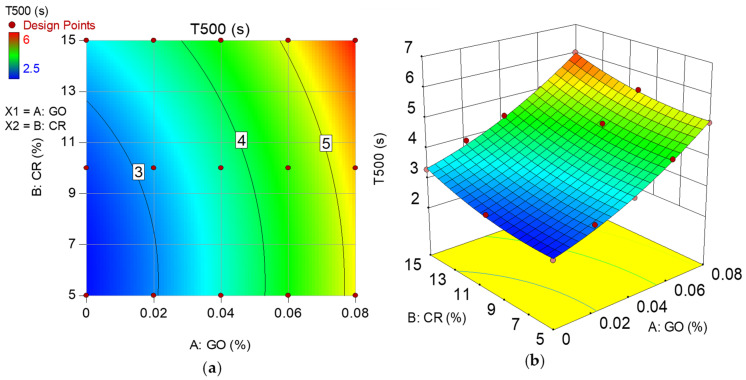
(**a**) 2D contour plot and (**b**) 3D response surface diagram for T_500_.

**Figure 9:**
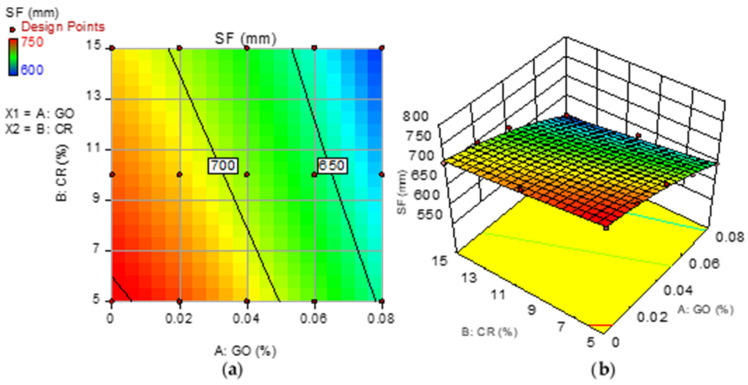
(**a**) 2D contour plot and (**b**) 3D response surface diagram for SF.

**Figure 10:**
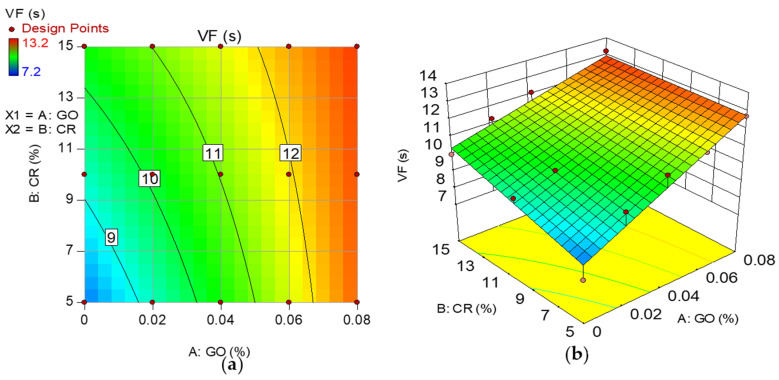
(**a**) 2D contour plot and (**b**) 3D response surface diagram for VF.

**Figure 11:**
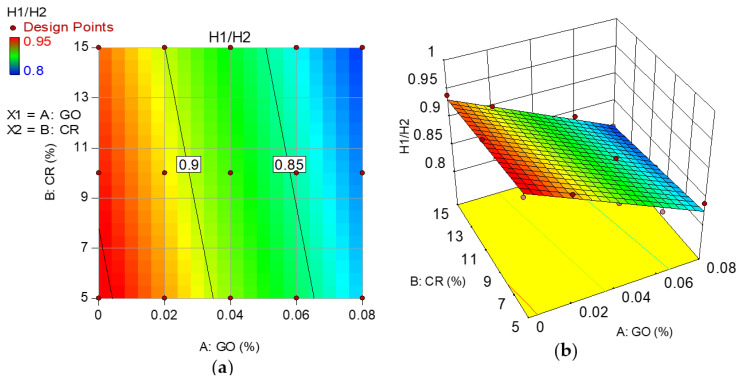
(**a**) 2D contour plot and (**b**) 3D response surface diagram for H2/H1.

Model graphs in the form of 2D contour and 3D response surface diagrams depict the individual and combined effects of the input factors and their interactions on the response. The graphs are represented by contours, which depict response changes at various degrees of input factors as shown in Figure 8, Figure 9, Figure 10 and Figure 11, respectively, for the developed models. A color gradient depicts the magnitude of the responses, with red portions representing the most significant values and blue sections suggesting the lowest values. The model graphs agree with the earlier findings discussed for the self-compacting properties.

**Figure 12:**
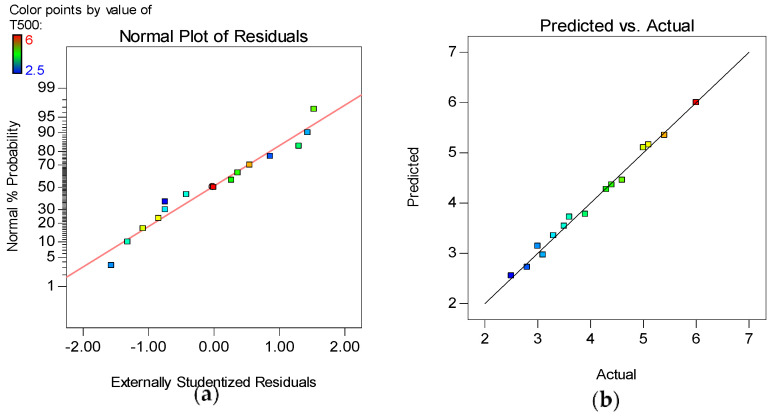
(**a**) Normal plot of residuals and (**b**) Actual vs. Predicted plot for T_500_.

**Figure 13:**
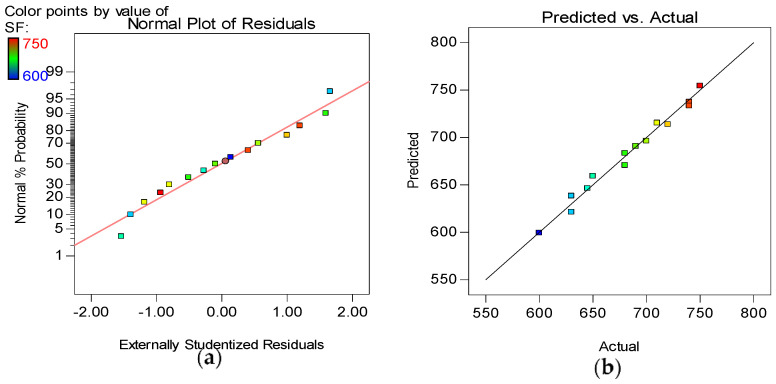
(**a**) Normal plot of residuals and (**b**) Actual vs. Predicted plot for SF.

**Figure 14:**
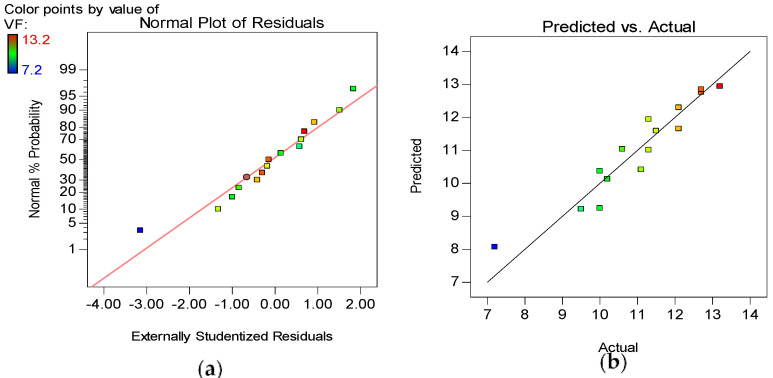
(**a**) Normal plot of residuals and (**b**) Actual vs. Predicted plot for VF.

**Figure 15:**
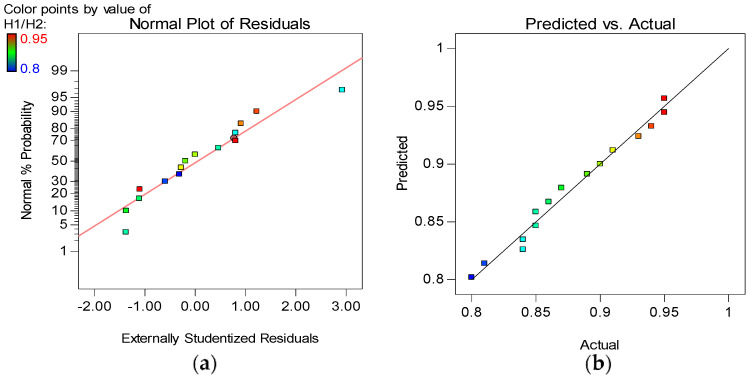
(**a**) Normal plot of residuals and (**b**) Actual vs. Predicted plot for H1/H2.

The model diagnostic tools used include the Normal plot of Residuals and the Actual versus Predicted graphs, as shown in Figure 12, Figure 13, Figure 14 and Figure 15 for all the developed models. The normality plot checks if the residuals are normally distributed, as seen by the data points’ linearity. In this instance, the accuracy of all generated models is demonstrated by data points aligning along the straight line. Similarly, by the data points of all the models following the 45° line in the Predicted versus Actual plots, the reliability of the models in predicting the responses with high accuracy is confirmed.

### 3.6. Optimization

The optimization aims to find acceptable values of the independent variables to achieve an optimal level of the desired response. This is accomplished by assigning different criteria and levels of significance to the variables (input factors and response) to fulfill the objective function. The desirability value (0 ≤ dj ≤ 1) is used to assess the optimization. The better the outcome (often stated as a percentage), the closer the number is to 1 [1].

**Figure 16:**
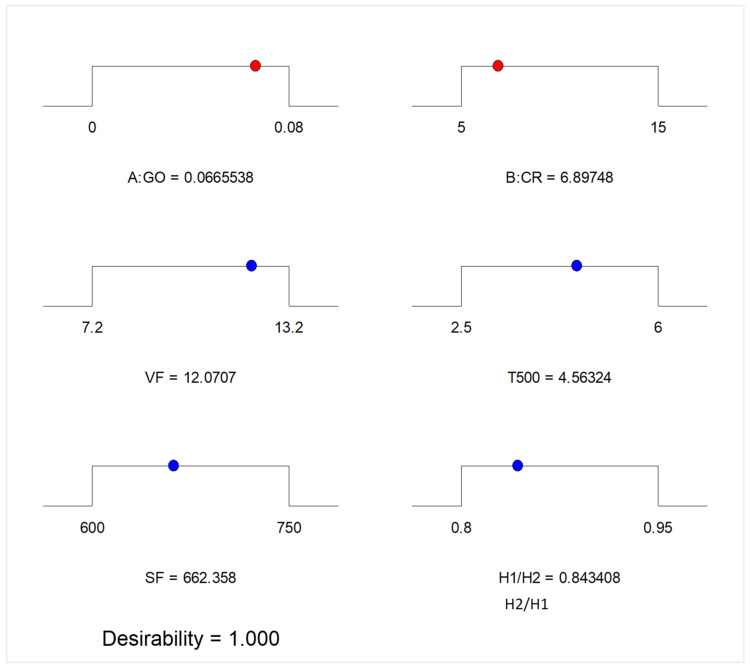
Optimization solution.

The optimization criteria, in this case, are presented in Table 4. The objectives for all the variables were set to be “In range” such that the system could choose the most suitable levels of the input factors from the lower and upper values that could yield the most optimum responses. After running the optimization, the solutions obtained are presented in the form of ramps in Figure 16. The optimum levels of the input factors were obtained as 0.067 wt.% and 6.90% for the GO and CR, respectively. The optimum responses predicted at the levels of the optimum input factors were 4.56 s, 662.36 mm, 12.07 s, and 0.843 for the T_500_, Sf, VF, and H2/H1, respectively. The optimization was done at the desirability of 1.0, indicating an excellent solution. These response values are within the specified limits by EFNARC.

### 3.7. Experimental Validation

Usually, the final step of RSM analysis involves the conduct of experimental investigations with the view to validate the predictive models and the optimization. A mix containing the optimum amount of the input variables was made, and all the workability tests were performed. The predicted and the experimental results are presented in Table 5. In addition, the percentage error between the experimental and the predicted results calculated from Equation (7) is also presented in Table 5. As can be observed, there is a good agreement between the predicted and the experimental result by virtue of the experimental error values being well within acceptable limits. Hence, the developed models can be used to predict the responses with high accuracy.

(7) $$\mathrm{Experimental}\;\mathrm{error}\;(\delta )=\left|\frac{Experimental\;value-Predicted\;value}{Predicted\;value}\right|\times 100\%$$

## 4. Conclusions

This research was done to assess GO and CR’s combined effect on SC-ECC’s workability properties and to develop predictive models of the properties using RSM, and the following conclusions were drawn:

1. The use of GO and CR together affects ECC’s self-compacting properties by lowering the flowability of the mixes caused by the GO particles’ high water absorption due to internal curing and the rough surface texture and lower density of the CR particles;
2. Despite the adverse effects of the GO and CR on the fluidity of the ECC mixes, all the self-compacting properties were found to be within acceptable limits set by EFNARC 2005. Hence, this confirms that the presence of GO and CR does not hinder the attainment of the desired SC characteristics. However, better performance of the fresh ECC mixes was attained at lower levels of the input factors (GO and CR);
3. Response surface models developed to predict the SC-GO-RECC’s self-compacting properties were confirmed to be very accurate with a coefficient of determination (R^2^) between 91–99% for all the SC parameters considered;
4. GO addition of 0.067 wt.%, and CR replacement of 6.8% was obtained as the optimum levels of the input variables generated by the optimization, which will guarantee an SC-GO-RECC with the best performance in the fresh state;
5. As a limitation, the results presented and the models developed are only applicable and valid within the limits of the input factors considered in this study which are 0 to 0.08 wt.% of cement for GO and 5% to 10% for CR replacement by volume of fine aggregate.

## Figures and Tables

**Table 1:** RSM generated runs, and the proportions materials.

Run (Mix)	Input Factors		PVA (%)	FA (%)	Quantities of Materials (kg/m^3^)						
	A: GO (%)	B: CR (%)			GO	CR	PVA	FA	Cement	Sand	Water
NECC	0	0	1.75	55	0	0	22.75	705.65	577.35	466.6	320
M1	0.02	10	1.75	55	0.115	3.9	22.75	705.65	577.35	463.1	320
M2	0.08	10	1.75	55	0.462	3.9	22.75	705.65	577.35	463.1	320
M3	0.08	5	1.75	55	0.462	1.3	22.75	705.65	577.35	465.7	320
M4	0.04	15	1.75	55	0.231	6.5	22.75	705.65	577.35	460.5	320
M5	0.02	15	1.75	55	0.115	6.5	22.75	705.65	577.35	460.5	320
M6	0	10	1.75	55	0	3.9	22.75	705.65	577.35	463.1	320
M7	0.04	10	1.75	55	0.231	3.9	22.75	705.65	577.35	463.1	320
M8	0.04	5	1.75	55	0.231	1.3	22.75	705.65	577.35	465.7	320
M9	0	15	1.75	55	0	6.5	22.75	705.65	577.35	460.5	320
M10	0.06	15	1.75	55	0.346	6.5	22.75	705.65	577.35	460.5	320
M11	0.06	10	1.75	55	0.346	3.9	22.75	705.65	577.35	463.1	320
M12	0.02	5	1.75	55	0.115	1.3	22.75	705.65	577.35	465.7	320
M13	0.08	15	1.75	55	0.462	6.5	22.75	705.65	577.35	460.5	320
M14	0	5	1.75	55	0	1.3	22.75	705.65	577.35	465.7	320
M15	0.06	5	1.75	55	0.346	1.3	22.75	705.65	577.35	465.7	320

**Table 2:** Summary of ANOVA.

Response	Source	Sum of Squares	Df	Mean Square	F-Value	*p*-Value > F	Significance
T_500_ (s)	Model	15.05	5	3.01	226.03	<0.0001	YES
	A-GO	12.94	1	12.94	971.19	<0.0001	YES
	B-CR	1.68	1	1.68	126.20	<0.0001	YES
	AB	5 × 10^−4^	1	5.0	0.038	0.8507	NO
	A^2^	0.26	1	0.26	19.47	0.0017	YES
	B^2^	0.18	1	0.18	13.24	0.0054	NO
	Residual	0.12	9	0.013			
	Cor Total	15.17	14				
SF (mm)	Model	28,636.67	5	5727.33	100.41	<0.0001	YES
	A-GO	23,520.00	1	23,520.00	412.36	<0.0001	YES
	B-CR	4622.50	1	4622.50	81.04	<0.0001	YES
	AB	20.00	1	20.00	0.35	0.5683	NO
	A^2^	466.67	1	466.67	8.18	0.0188	YES
	B^2^	7.50	1	7.50	0.13	0.7253	NO
	Residual	513.33	9	57.04			
	Cor Total	29,150.00	14				
VF(s)	Model	29.91	3	9.97	36.03	<0.0001	YES
	A-GO	24.66	1	24.66	89.13	<0.0001	YES
	B-CR	3.84	1	3.84	13.89	0.0033	YES
	AB	1.40	1	1.40	5.08	0.0456	YES
	Residual	3.04	11	0.28			
	Cor Total	32.95	14				
H2/H1	Model	0.033	2	0.017	313.63	<0.0001	YES
	A-GO	0.032	1	0.032	600.25	<0.0001	YES
	B-CR	1.4 × 10^−3^	1	1.4 × 10^−3^	27.00	0.0002	YES
	Residual	6.4 × 10^−4^	12	5.3 × 10^−5^			
	Cor Total	0.034	14				

**Table 3:** Model validation Parameters.

Parameters	T_50__0_ (s)	Slump Flow (mm)	V-Funnel (s)	L-Box Ratio (H2/H1)
Standard Dev.	0.12	7.55	0.53	7.30 × 10^−3^
Mean	4.03	685.00	11.03	0.88
C.V. %	2.86	1.10	4.77	0.83
PRESS	0.29	1401.60	6.97	1.095 × 10^−3^
−2Log Likelihood	−29.87	95.56	18.64	−108.36
R^2^	0.9921	0.9824	0.9076	0.9812
Adjusted R^2^	0.9877	0.9726	0.8825	0.9781
Predicted R^2^	0.9806	0.9519	0.7886	0.9679
Adeq. Precis.	47.219	32.451	17.917	47.357
BIC	−13.62	111.81	29.47	−100.24
AIC	−7.37	118.06	30.64	−100.18

**Table 4:** Optimization criteria and goals.

Factors/Response	Unit	Goal	Lower Limit	Upper Limit
GO	%	In range	0	0.08
CR	%	In range	5	15
T_500_	s	In range	2.5	6
SF	mm	In range	600	750
VF	s	In range	7.2	13.2
H2/H1	-	In range	0.8	0.95

**Table 5:** Experimental validation and percentage error values.

Response	Predicted	Experimental	δ (%)
T_500_ (s)	4.56	4.33	5.0
SF (mm)	662.36	700	5.6
VF (s)	12.07	11.11	8.0
H2/H1	0.84	0.88	4.7

## Data Availability

All data are contained in the paper.
